# COGNITIVE HEALTH AND ADRD: GENDER, RACE, AND ETHNIC ISSUES

**DOI:** 10.1093/geroni/igz038.1995

**Published:** 2019-11-08

**Authors:** Toni C Antonucci, Kristine Ajrouch, Jonathan W King

**Affiliations:** 1 University of Michigan, Ann Arbor, Michigan, United States; 2 Eastern Michigan University, Ypsilanti, Michigan, United States; 3 National Institute on Aging, Bethesda, Maryland, United States

## Abstract

This symposium addresses minority issues in aging, specifically issues of gender, race and ethnicity, in the study of cognitive health and Alzheimer’s Disease and Related Dementia (ADRD). Cabrera examines ADRD in the Latino community noting that there are or may be important differences among subgroups of Latinos, e.g. Mexicans and Puerto Ricans, who are too often considered a homogenous group. Using qualitative methods, specifically focus groups, she explores whether these two groups have a different understanding of or different concerns about ADRD. Dallo considers the epidemiology of ADRD among Arab Americans. Noting the dearth of evidence on this ethnic group, she uses the National Health Interview Survey from 2000-2017 to examine the prevalence of ADRD among foreign-born Arab American compared to Whites, Blacks and Asians. Indiro et al., consider the long reach of childhood SES on age-related brain changes in different racial and ethnic older adults. Finally, Byrd considers gender differences in cognitive health in the Baltimore Study of Black Aging and finds that women actually report better cognitive health than men, controlling for age, education and health status despite previous literature suggesting that women experience more dementia than men. In total, this symposium highlights the need to consider contexts such as gender, race and ethnicity in order to fully understand factors influencing cognitive health and ADRD

